# Skeletal Muscle Radiation Attenuation at C3 Predicts Survival in Head and Neck Cancer

**DOI:** 10.3390/curroncol32100587

**Published:** 2025-10-21

**Authors:** Felix Barajas Ordonez, Kunpeng Xie, André Ferreira, Robert Siepmann, Najiba Chargi, Sven Nebelung, Daniel Truhn, Stefaan Bergé, Philipp Bruners, Jan Egger, Frank Hölzle, Markus Wirth, Christiane Kuhl, Behrus Hinrichs-Puladi

**Affiliations:** 1 Department of Diagnostic and Interventional Radiology, University Hospital RWTH Aachen, 52074 Aachen, Germany; fbarajasordo@ukaachen.de (F.B.O.); rsiepmann@ukaachen.de (R.S.); snebelung@ukaachen.de (S.N.); dtruhn@ukaachen.de (D.T.); pbruners@ukaachen.de (P.B.); ckuhl@ukaachen.de (C.K.); 2 Department of Oral and Maxillofacial Surgery, University Hospital RWTH Aachen, 52074 Aachen, Germany; id10656@alunos.uminho.pt (A.F.); fhoelzle@ukaachen.de (F.H.); bpuladi@ukaachen.de (B.H.-P.); 3 Institute of Medical Informatics, University Hospital RWTH Aachen, 52074 Aachen, Germany; 4 Institute for AI in Medicine (IKIM), University Hospital Essen (AöR), 45131 Essen, Germany; 5 Center Algoritmi/LASI, University of Minho, 4710-057 Braga, Portugal; 6 Department of Oral and Maxillofacial Surgery, Radboud University Medical Center, 6525 GA Nijmegen, The Netherlands; chargi.najiba@gmail.com (N.C.); stefaan.berge@radboudumc.nl (S.B.); 7 Department of Otolaryngology Head and Neck Surgery, University Hospital RWTH Aachen, 52074 Aachen, Germany; mwirth@ukaachen.de

**Keywords:** head and neck neoplasms, skeletal muscle, myosteatosis, survival analysis, tomography

## Abstract

**Background**: Sarcopenia assessed by skeletal muscle area (SMA) at the third lumbar vertebra (L3) is an established prognostic marker in many malignancies, including head and neck cancer (HNC). However, in HNC, L3 is rarely assessed. The prognostic value of myosteatosis, measured by skeletal muscle radiation attenuation (SMRA) remains largely unexplored. This study evaluated both muscle metrics at the third cervical vertebra (C3) for locoregional control (LRC) and overall survival (OS) in HNC. **Methods**: SMA and SMRA at C3 were quantified in CT scans of 904 HNC cases by a deep learning-based segmentation pipeline with manual verification. Cox proportional hazards models assessed associations with LRC and OS. **Results**: Median SMA was 36.64 cm^2^ (IQR: 30.12–42.44). Median SMRA was 50.77 HU (IQR: 43.04–57.39). In multivariable analysis, lower SMA (HR 1.85, 95% CI: 1.19–2.88, *p* ≤  0.001) and lower SMRA (HR 1.76, 95% CI: 1.22–2.54, *p* < 0.001) were associated with lower LRC. For OS, lower SMA (HR 1.53, 95% CI:1.06–2.20, *p* = 0.02) and lower SMRA (HR 2.13, 95% CI: 1.58–2.88, *p* <  0.001) were associated with a worse outcome in multivariable analysis. **Conclusions**: Both SMRA and SMA assessed at C3 correlate with worse LRC and OS in HNC.

## Simple Summary

Patients with head and neck cancer (HNC) are at high risk of malnutrition due to tumor location and treatment. Imaging performed for tumor evaluation can also provide information about a patient’s body composition. Most existing data are based on measurements at the lumbar spine. In contrast, fewer data are available at the cervical spine . In this study, we applied an automated radiological assessment to routine neck CT scans to measure muscle quantity and muscle quality at C3. Poor muscle quality was identified as a stronger predictor of patient outcomes than reduced muscle quantity. These findings support existing data that head and neck imaging can evaluate muscle quality, thereby providing valuable prognostic information regarding nutritional status, and correlating with shorter survival and poor local treatment response.

## 1. Introduction

Head and neck (H&N) imaging is routinely performed in head and neck cancer (HNC) management, guiding diagnosis, staging, treatment, and follow-up [1,2,3,4]. In addition to traditional clinical and pathological prognostic factors, emerging evidence shows that radiologically defined sarcopenia is also associated with worse oncological outcomes in HNC, relying largely on skeletal muscle area (SMA) assessment at the third lumbar vertebra (L3) [5,6,7]. A recent systematic review by Syziu and Schache (2025) [7] showed that pre-treatment sarcopenia at either L3 or the third cervical vertebra (C3) was associated with worse overall survival (OS) in 26 of the 30 studies (87%). Likewise, van Heusden et al. (2025) [6], in a meta-analysis of 63 studies (14,804 patients), reported that sarcopenia significantly increased the risk of both impaired OS (log OR 0.808, 95% [confidence interval] CI 0.509–1.107, *p* < 0.001) and treatment-related complications (log OR 0.669, 95% CI 0.441–0.897, *p* < 0.001). While L3 remains the most widely used reference level due to established cut-off values, it is rarely available in HNC. By contrast, C3 is almost always included in H&N scans, and its use has also been reported. Erul et al. (2023) [8] reported that 66 of 113 HNC patients undergoing cisplatin-based chemoradiation (CRT) had pre-treatment sarcopenia assessed by sternocleidomastoid muscle volume at the C3 level, and this was associated with worse OS (hazard ratio [HR] 2.86; 95% CI: 1.40–5.85; *p* = 0.004).

Few studies have explored the prognostic value of muscle quality, rather than quantity. Shaver et al. (2022) [9] demonstrated, in 403 patients treated with radiotherapy (RT), that myosteatosis, defined by low skeletal muscle density (SMD) at L3, was independently associated with worse OS HR 1.55 (95% CI 1.03–2.34). Similarly, Bardoscia et al. (2022) [10] analyzed 225 patients undergoing CRT and found that low SMD at L3 independently predicted worse OS and progression-free survival (PFS). Evidence on myosteatosis in HNC, however, is still scarce and largely based on L3-based analyses. This study evaluated whether skeletal muscle radiation attenuation (SMRA) at C3 level provides stronger prognostic information than SMA for locoregional control (LRC) and OS in HNC.

## 2. Materials and Methods

### 2.1. Study Design

A total of 904 of an initial 1355 HNC cases, with M0 disease and an evaluable C3 area on H&N CT scans, and complete clinical variables apart from HPV status were screened and selected from four public databases hosted on The Cancer Imaging Archive (TCIA): Head-Neck-PET-CT [11], TCGA-HNSC [12], CPTAC-HNSCC [13], and HEAD-NECK-RADIOMICS-HN1 [14]. Clinical variables retrieved included age, sex, tumor localization, TNM classification, human papillomavirus (HPV) status if available, therapy type, and prior surgery. For cases with cancer of unknown primary (CUP), a tumor stage of T0 was assigned. Outcome variables extracted included OS and LRC (assessed as time to locoregional recurrence).

### 2.2. Deep Learning Pipeline for SMA and SMRA Quantification

A two-step approach was used to detect and segment the skeletal muscle at C3. First, a detection model was trained to generate a bounding box centered at C3 and to extract the middle slices. In the middle slice, a segmentation model extracted the skeletal muscle mask, including the paravertebral muscles and the sternocleidomastoid muscles bilaterally, from which SMA (cm^2^) and SMRA (HU) were calculated.

### 2.3. Automatic Detection

From a randomly selected subset of 100 cases (from the 904), bounding boxes at the C3 level were manually annotated to train a customized automatic C3 detector. Based on the high performance of RetinaNet in the LUNA16 dataset (https://luna16.grand-challenge.org/Data/, accessed on 7 March 2024), this network was trained in the MONAI framework (https://github.com/Project-MONAI/tutorials/tree/main/detection, accessed on 7 March 2024) for 3D detection. A smooth L1 loss function and stochastic gradient descent were used with a learning rate of 1 × 10^−2^ and a weight decay of 3 × 10^−5^. Training was performed using 5-fold cross-validation for 300 epochs. A visual inspection was subsequently performed by a radiology resident with four years’ experience to ensure accurate segmentation of the musculature and the exclusion of other structures, such as lymph nodes. Manual adjustments were then performed before data extraction.

### 2.4. Automatic Segmentation

For the segmentation of the C3 slices selected in the prior step, a deep learning (DL) model pre-trained for the segmentation of muscle in C3 slices was used. This model is available in the repository (https://github.com/xmuyzz/C3-Segmentation, accessed on 7 March 2024). Segmentation results were reviewed by a radiology resident experienced in segmentation techniques; cases with inaccuracies in C3 detection or segmentation were re-evaluated manually, correcting C3 slice selection and segmentation as needed. Results were reviewed and finalized to ensure quality control. This comprehensive pipeline combined automation with manual validation to optimize accuracy and reproducibility in the detection and segmentation of the C3 vertebral region.

### 2.5. Statistical Analysis

The distribution of continuous variables was assessed visually using histograms and tested for normality using the Shapiro–Wilk and Kolmogorov–Smirnov tests. Variables with a non-normal distribution were presented as medians with interquartile ranges (IQR; 25th–75th percentiles). Those with approximately normal distributions were presented as means with standard deviations (SD). Kaplan–Meier survival analysis was used to estimate LRC and OS, with survival curves compared across body composition groups using the log-rank test to identify statistically significant differences. Cox proportional hazards regression models were applied to evaluate LRC and OS, calculating HRs with 95% CI and *p*-values. The multivariable models were adjusted for key prognostic factors in HNC, including age (≤70 vs. >70) [15], sex, T stage (T0–T2 vs. T3–T4), N stage (N0–N1 vs. N2–N3), and therapy type (RT alone, surgery + RT, or concurrent CRT), while simultaneously including SMA and SMRA as dichotomous traits. All multivariable Cox models were fitted on a complete-case basis. Optimal cut-off values were explored using three approaches: (i) the 25th percentile, (ii) the 33rd percentile (tertiles), and (iii) receiver operating characteristic (ROC) curve-derived Youden indices for 60-month outcomes (OS and LRC). First-order interaction terms between SMA or SMRA and sex or T stage were additionally evaluated within the Cox models, and significance was assessed using Wald tests. Model discrimination was assessed using Harrell’s c-index with 95% confidence intervals, and the proportional hazards assumption was tested using Schoenfeld residuals. For sensitivity analyses, multivariable Cox models were fitted in the subset of patients with oropharyngeal carcinoma and documented HPV status. Statistical significance was defined as *p* < 0.05, and all analyses were performed using R (version 4.5.1; R Foundation for Statistical Computing, Vienna, Austria).

## 3. Results

### 3.1. Patient Characteristics (Supplementary Table S1)

Of 904 patients, median age was 59 years (IQR: 53–66 years) and 83% were men (*n* = 748 patients). Tumor localization was primarily oropharynx (82%, *n* = 738), larynx (7%, *n* = 66), nasopharynx (4%, *n* = 34), hypopharynx (3%, *n* = 27), CUP (4%, *n* = 32), and oral cavity (1%, *n* = 7). T-stage: T1 (17%, *n* = 157), T2 (37%, *n* = 334), T3 (26%, *n* = 237), and T4 (16%, *n* = 144). N-stage: N0 (13%, *n* = 116), N1 (12%, *n* = 106), N2 (including all subcategories, 70%, *n* = 635), and N3 (5%, *n* = 47). Overall stage: I (1%, *n* = 11), II (5%, *n* = 44), III (16%, *n* = 147), and IV (including all subcategories, 78%, *n* = 702). Treatment: RT alone (17%, *n* = 151), RT following surgery (4%, *n* = 33), CRT alone (67%, *n* = 607), and surgery then CRT (13%, *n* = 113).

### 3.2. Distribution of SMA and SMRA

The median SMA at C3 was 36.64 cm^2^ (IQR: 30.12–42.44 cm^2^). SMRA at C3 had a median value of 50.77 HU (IQR: 43.04–57.39 HU) (Supplementary Table S2a). For the dichotomization of the cohort, SMA (low vs. normal/high) and SMRA (low vs. normal/high) were defined using Youden-derived thresholds from 60-month LRCs (SMA ≤ 30.42 cm^2^, SMRA ≤ 50.72 HU), which showed the highest prognostic performance compared with percentile-based or OS-derived thresholds (Supplementary Tables S2b and S3a–d; Supplementary Figure S1).

### 3.3. Association Between SMA and SMRA Groups and LRC

Among body composition parameters, low SMA was significantly associated with poorer LRC in univariate analysis (HR 1.89, 95% CI 1.34–2.66, *p* < 0.001) and remained an independent predictor in the multivariable model (HR 1.85, 95% CI 1.19–2.88, *p* < 0.001). Similarly, low SMRA was strongly associated with locoregional failure in both univariate (HR 2.22, 95% CI 1.57–3.14, *p* < 0.001) and multivariate analyses (HR 1.76, 95% CI 1.22–2.88, *p* < 0.001) (Table 1). Model discrimination was moderate (c-index 0.68, 95% CI 0.63–0.72). No significant violation of proportional hazards was observed (global Schoenfeld test *p* = 0.76). No significant interactions with sex or T stage were observed for either SMA or SMRA (Supplementary Table S4a). In the oropharyngeal subset with known HPV status, low SMA (HR 1.51, 95% CI 0.63–3.66, *p* = 0.36) and low SMRA (HR 1.39, 95% CI 0.77–2.53, *p* = 0.28) did not retain significance (Supplementary Table S5).

### 3.4. Association Between SMA and SMRA Groups and OS

**Figure 1:**
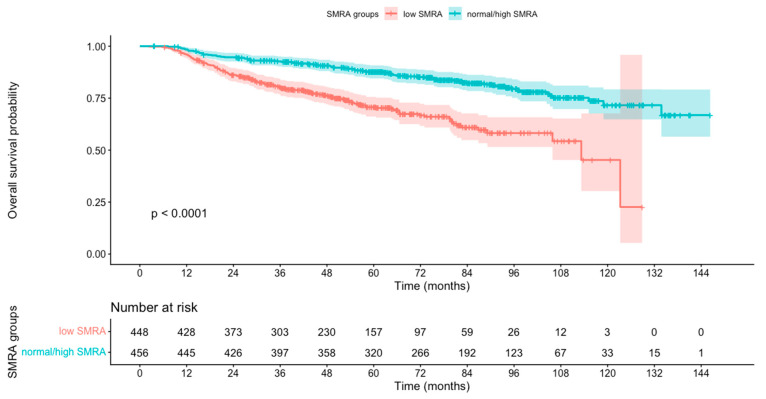
Kaplan–Meier survival analysis results for head and neck cancer (HNC) patients stratified by skeletal muscle radiation attenuation (SMRA) into low SMRA and normal/high SMRA groups.

Low SMRA was a predictor of worse OS, demonstrating significant associations in both univariate (HR 2.58, 95% CI 1.92–3.39, *p* < 0.001) and multivariable analyses (HR 2.13, 95% CI 1.58–2.88, *p* < 0.001). These findings were consistent with Kaplan–Meier analysis (Figure 1). Low SMA was also associated with reduced OS in univariate analysis (HR 1.46, 95% CI 1.09–1.96, *p* = 0.01) (Supplementary Figure S2) and in the multivariable model (HR 1.53, 95% CI 1.06–2.20, *p* = 0.02). Model discrimination was moderate (c-index 0.69, 95% CI 0.65–0.72). No significant violation of proportional hazards was observed (global Schoenfeld test *p* = 0.45). No significant interactions were observed between SMA or SMRA and sex or T stage, except for SMA × T stage (*p* = 0.02) (Supplementary Table S4b). In the subset with known HPV status, neither low SMA (HR 1.72, 95% CI 0.82–3.61, *p* = 0.15) nor low SMRA (HR 1.28, 95% CI 0.74–2.22, *p* = 0.37) retained prognostic significance (Supplementary Table S5).

## 4. Discussion

Our study demonstrated the feasibility and reproducibility of a DL-based segmentation pipeline to evaluate SMA and SMRA at the C3 level on CT as a risk assessment tool in HNC without requiring additional imaging acquisition. Our findings support previous evidence that muscle metrics correlate with worse oncological outcomes in HNC. Syziu and Schache (2025) [7] systematically reviewed 30 studies including 6924 patients and confirmed that pre-treatment sarcopenia at either L3 or C3 significantly predicted reduced OS. The meta-analysis by van Heusden et al. (2025) [6], pooling 63 studies with 14,804 patients, similarly demonstrated that sarcopenia significantly increased the risk of both impaired OS (log OR 0.808, 95% CI 0.509–1.107, *p* < 0.001) and treatment-related complications (log OR 0.669, 95% CI 0.441–0.897, *p* < 0.001). In summary, lower SMA was associated with worse LRC and OS, while lower SMRA, as a surrogate for myosteatosis, was a more robust predictor of both endpoints. A significant interaction between SMA and T stage (*p* = 0.02) indicated that the prognostic effect of SMA varied across tumor stages, whereas SMRA appeared less dependent on stage and more consistent overall, suggesting that muscle quality may have potential for integration into imaging-based risk stratification in HNC.

Most prior evidence relied on measurements at the L3 level [3,16,17], consistently reporting that sarcopenia at L3 before and after RT predicted decreased OS and PFS. Abdominal imaging is not, however, available in HNC. van Rijn-Dekker et al. [4] validated C3 as a surrogate for L3 in 750 patients with HNC, showing significant associations with OS and disease-free survival. Endo et al. [18] demonstrated that a low skeletal muscle index at C3, calculated by normalizing the SMA for height, predicted aspiration pneumonia in patients undergoing CRT; however, such anthropometric data were not available in our cohort, and we therefore analyzed absolute SMA. These findings establish C3 as a clinically relevant anatomical level for muscle assessment in HNC. In our subgroup analysis of oropharyngeal carcinoma cases with documented HPV status, the associations between muscle metrics and survival outcomes were no longer significant. This likely reflects the overrepresentation of HPV-positive tumors among the reported cases, as HPV status was available for less than half of the cohort. Future studies with more balanced HPV data are warranted to confirm these observations.

Evidence regarding myosteatosis in HNC is scarce. Shaver et al. (2022) [9] demonstrated, in 403 patients treated with RT, that low SMD at the L3 was independently associated with worse OS (HR 1.55, 95% CI 1.03–2.34). Bardoscia et al. (2022) [10] reported, in 225 patients undergoing CRT, that low SMD at L3 independently predicted inferior OS and PFS. These studies underline the prognostic importance of muscle quality, relying on abdominal imaging. Our study extends this evidence by demonstrating that SMRA at C3 independently predicts OS and LRC, thereby providing prognostic information at the anatomical level consistently available in H&N imaging. These results are biologically plausible, as skeletal muscle serves as an energy reservoir that is mobilized during cancer as a catabolic state, which is further aggravated by chemotherapy [19]. Intramuscular adipose tissue infiltration (myosteatosis) may contribute to disease progression through the local secretion of inflammatory adipokines from adipocytes, which induce insulin resistance, impairs insulin diffusion capacity, and weakens immune defenses. This pro-inflammatory and metabolic dysregulation is consistent with prior observations linking myosteatosis to systemic inflammation and adverse metabolic signaling in solid tumors [20,21].

In a meta-analysis by Schaeffers et al. [5] including 22 studies, skeletal muscle mass was identified as a biomarker predicting dose-limiting toxicity (DLT) during CRT in HNC. Although DLT was not evaluated in our study, the role of myosteatosis compared with sarcopenia is also an important subject for further research. Since myosteatosis may occur in the absence of sarcopenia, it may provide complementary prognostic information beyond muscle quantity alone [20]. Patients with markedly reduced SMRA may be at increased risk of malnutrition, high recurrence rates, and potential treatment intolerance. Although prospective validation is required, early nutritional assessment based on C3-derived muscle evaluation could help identify high-risk patients in whom, for instance, prophylactic gastrostomy placement may prevent therapy-related complications. Furthermore, because L3 is not routinely covered in H&N follow-up imaging, C3 measurements may provide valuable longitudinal information at interim time points and enable intraindividual cross-sectional comparisons. Such proactive management carries minimal risk and could be clinically beneficial. In addition, DL-based assessments could facilitate standardized SMRA quantification and support individualized care by helping to triage patients for nutritional optimization, disease stage-specific interventions, or treatment-intensity adaptations within multidisciplinary workflows.

### 4.1. Limitations

As H&N CT imaging is routinely performed in HNC patients, C3-based assessment is a feasible and promising approach for integrating muscle quality and quantity into oncologic risk stratification. Our study is, however, limited by the lack of established cutoff values for SMA and SMRA. Validated values are needed to standardize these parameters for clinical application. Anthropometric parameters might also be of interest. Additional limitations include the absence of treatment dose/RT plan information, scanner/protocol heterogeneity across TCIA datasets, and potential selection bias from cases with incomplete C3 coverage.

### 4.2. Future Perspective

In HNC, the prognostic impact of the longitudinal SMA, and especially, SMRA assessment remains less well-established than measurements at L3 in other malignancies. Future research in HNC should focus on validating standardized cutoffs at C3, assessing its predictive value for treatment toxicity and therapy response, and exploring whether dynamic changes in SMRA during treatment provides prognostic value beyond baseline assessments.

## 5. Conclusions

Our findings indicate that the DL-supported measurement of SMA and SMRA at the C3 level is feasible, reproducible, and applicable for body composition assessment in HNC. Specifically, SMRA serves as a robust biomarker and a surrogate for myosteatosis, with low SMRA independently associated with an increased risk of locoregional recurrence and mortality. These findings reinforce their potential role in oncologic risk stratification and highlight the importance of muscle quality over muscle quantity in prognostic assessment. Validated values are needed to standardize parameters for clinical application, but the integration of automated SMRA quantification into routine imaging workflows offers a scalable, non-invasive, and time-efficient method for risk assessment, without requiring additional imaging.

## Figures and Tables

**Table 1:** Cox proportional hazards models for oncological outcomes by SMA and SMRA (as dichotomous traits) (*n* = 904).

	Univariate Analysis			Multivariable Analysis *		
Variable	HR	95% CI	*p*-Value	HR	95% CI	*p*-Value
Locoregional control						
SMRA	2.22	[1.57–3.14]	<0.001	1.76	[1.22–2.54]	<0.001
SMA	1.89	[1.34–2.66]	<0.001	1.85	[1.19–2.88]	<0.001
Overall survival						
SMRA	2.58	[1.92–3.39]	<0.001	2.13	[1.58–2.88]	<0.001
SMA	1.46	[1.09–1.96]	0.01	1.53	[1.06–2.20]	0.02

Hazard ratios (HRs) are calculated for low vs. normal/high in all models. CI, confidence interval; SMA, skeletal muscle area; SMRA, skeletal muscle radiation attenuation. * Multivariable models were additionally adjusted for age (≤70 vs. >70 years), sex, T stage (T0–T2 vs. T3–T4), N stage (N0–N1 vs. N2–N3), and therapy (RT alone vs. CRT).

## Data Availability

The data presented in this study are available on reasonable request.

## Supplementary Materials

The following supporting information can be downloaded at: https://www.mdpi.com/article/10.3390/curroncol32100587/s1, Figure S1: (a) Time-dependent receiver operating characteristic (ROC) curves at 60 months for skeletal muscle area (SMA, cm^2^) and skeletal muscle radiation attenuation (SMRA, HU) at cervical level C3, predicting locoregional control (LRC), (b) Time-dependent receiver operating characteristic (ROC) curves at 60 months for skeletal muscle area (SMA, cm^2^) and skeletal muscle radiation attenuation (SMRA, HU) at cervical level C3, predicting overall survival (OS); Figure S2: Kaplan-Meier survival analysis results for head and neck cancer (HNC) patients stratified by skeletal muscle area (SMA) into low and normal/high SMA groups; Table S1: Patient characteristics (*n* = 904); Table S2: (a) Measurements of SMA and SMRA at Cervical Level C3 (*n* = 904); (b) Cut-off values for SMA and SMRA at the cervical level C3 for predicting 60-month OS and LRC (*n* = 904); Table S3: (a) Cox proportional hazards models for oncological outcomes by SMA and SMRA analyzed as dichotomous variables based on percentile-derived cut-offs (≤25%) (*n* = 904); (b) Cox proportional hazards models for oncological outcomes by SMA and SMRA, analyzed as dichotomous variables based on tertile-derived cut-offs (≤33%) (*n* = 904); (c) Cox proportional hazards models for oncological outcomes by SMA and SMRA, analyzed as dichotomous variables based on Youden-index–derived cut-offs for LRC at 60 months (*n* = 904); (d) Cox proportional hazards models for oncological outcomes by SMA and SMRA, analyzed as dichotomous variables based on Youden-index–derived cut-offs for OS at 60 months; Table S4: (a) Interaction tests of SMA and SMRA (dichotomized by Youden cut-off for 60-month LRC) with sex and T stage in multivariable Cox regression for LRC; (b) Interaction tests of SMA and SMRA (dichotomized by Youden cut-off for 60-month LRC) with sex and T stage in multivariable Cox regression for OS; Table S5: Cox proportional hazards models for oncologic outcomes according to SMA and SMRA (as dichotomous variables) in oropharyngeal carcinoma cases with documented HPV status (*n* = 381).

## Abbreviations

C3	Third Cervical Vertebra
CI	Confidence Interval
CRT	Chemoradiation
CUP	Cancer of Unknown Primary
CSA	Cross-Sectional Area
DL	Deep Learning
DLT	Dose-Limiting Toxicity
H&N	Head and Neck
HNC	Head and Neck Cancer
HPV	Human Papillomavirus
HR	Hazard Ratio
L3	Third Lumbar Vertebra
LRC	Locoregional Control
OS	Overall Survival
PFS	Progression-Free Survival
RT	Radiotherapy
SMA	Skeletal Muscle Area
SMD	Skeletal Muscle Density
SMRA	Skeletal Muscle Radiation Attenuation
