# Supplementary material for: Skeletal Muscle Radiation Attenuation at C3 Predicts Survival in Head and Neck Cancer
Source: Curr Oncol. 2025 Oct 21;32(10):587. doi: 10.3390/curroncol32100587 (PMC12562968; doi:10.3390/curroncol32100587)
Supplement: Supplementary file 1 [file curroncol-32-00587-s001.zip › curroncol-3815134-supplementary.pdf]

Supplementary materials

Supplementary tables

Table S1. Patient characteristics (n = 904).

**Variable**		***n (%)***
**Age, median (IQR)**		59.0 (53.0 – 66.0)
**Sex**	Female	156 (17.3)
	Male	748 (82.7)
**Tumor Localization**	Oropharynx	738 (81.6)
	Larynx	66 (7.3)
	CUP	32 (3.5)
	Nasopharynx	34 (3.8)
	Hypopharynx	27 (3.0)
	Oral Cavity	7 (0.8)
**TNM classification**		
T	T0	32 (3.5)
	T1	157 (17.4)
	T2	334 (36.9)
	T3	237 (26.2)
	T4 (T4a and T4b)	144 (15.9)
N	N0	116 (12.8)
	N1	106 (11.7)
	N2 (2a, 2b and 2c)	635 (70.2)
	N3	47 (5.2)
M	0	904 (100)
	1	0 (0)
**Stage**	Stage I	11 (1.2)
	Stage II	44 (4.9)
	Stage III	147 (16.3)
	Stage IV (IVA and IVB)	702 (77.6)
**HPV status**	Positive	333 (36.8)
	Negative	100 (11.1)
	Unknown	471 (52.1)
**Therapy**	Radiotherapy alone	151 (16.7)
	Surgery + RT alone	33 (3.7)
	Concurrent RTC	607 (67.1)
	Surgery + RTC	113 (12.5)

IQR, interquartile range; CUP, Cancer of Unknown Primary, HPV (Human Papillomavirus); RT, radiotherapy; CRT, chemoradiation.

Table S2a. Measurements of SMA and SMRA at Cervical Level C3 (n = 904).

**Parameter**	25th Percentile	33rd Percentile	Median	66th Percentile	75th Percentile
SMA (cm^2)	30.12	32.48	36.64	40.08	42.44
SMRA (HU)	43.04	45.85	50.77	55.16	57.39

SMA, skeletal muscle area; SMRA, skeletal muscle radiation attenuation

Table S2b. Cut-off values for SMA and SMRA at the cervical level C3 for predicting 60-month OS and LRC (n = 904).

Parameter	AUC (OS, 60m)	Cut-off (cm^2/HU)	Sens	Spec	AUC (LRC, 60m)	Cut-off (cm^2/HU)	Sens	Spec
SMA	0.58	40.86 cm^2	0.80	0.33	0.59	30.42 cm^2	0.38	0.76
SMRA	0.63	50.59 HU	0.68	0.55	0.63	50.72 HU	0.70	0.54

Youden-derived cut-offs for skeletal muscle area (SMA) and skeletal muscle radiation attenuation (SMRA) at cervical level C3 for overall survival (OS) and locoregional control (LRC) at 60 months. AUC, area under the curve; HU, Hounsfield unit; Sens, sensitivity; Spec, specificity.

Table S3a. Cox proportional hazards models for oncological outcomes by SMA and SMRA, analyzed as dichotomous variables based on percentile-derived cut-offs (≤25%). (n = 904).

	**Multivariable analysis***		
**Variable**	**HR**	**95% CI**	**p-value**
Locoregional control			
SMRA percentile (≤25%)	1.89	[1.28 – 2.77]	< 0.001
SMA percentile (≤25%)	1.62	[1.02 –2.58]	0.04
Overall survival			
SMRA percentile (≤25%)	2.10	[1.52 – 2.91]	< 0.001
SMA percentile (≤25%)	1.39	[0.95 – 2.04]	0.09

Hazard ratios (HR) are calculated for low vs. normal/high in all models. CI, confidence interval; SMA, skeletal muscle area; SMRA, skeletal muscle radiation attenuation.

* Multivariable models were additionally adjusted for age (≤70 vs. >70 years), sex, T stage (T0–T2 vs. T3–T4), N stage (N0–N1 vs. N2–N3), and therapy (RT alone vs. CRT)

Table S3b. Cox proportional hazards models for oncological outcomes by SMA and SMRA, analyzed as dichotomous variables based on tertile-derived cut-offs (≤33%) (n = 904).

	**Multivariable analysis***		
**Variable**	**HR**	**95% CI**	**p-value**
Locoregional control			
SMRA tertile (≤33%)	1.60	[1.12 –2.29]	0.01
SMA tertile (≤33%)	1.34	[0.89–2.03]	0.16
Overall survival			
SMRA tertile (≤33%)	1.94	[1.44–2.62]	0.001
SMA tertile (≤33%)	1.21	[0.87 – 1.69]	0.26

Hazard ratios (HR) are calculated for low vs. normal/high in all models. CI, confidence interval; SMA, skeletal muscle area; SMRA, skeletal muscle radiation attenuation.

* Multivariable models were additionally adjusted for age (≤70 vs. >70 years), sex, T stage (T0–T2 vs. T3–T4), N stage (N0–N1 vs. N2–N3), and therapy (RT alone vs. CRT)

Table S3c. Cox proportional hazards models for oncological outcomes by SMA and SMRA, analyzed as dichotomous variables based on Youden-index–derived cut-offs for LRC at 60 months (n = 904).

	Multivariable analysis*		
Variable	HR	95% CI	p-value
Locoregional control			
SMRA (Youden LRC)	1.76	[1.22 –2.54]	< 0.001
SMA (Youden LRC)	1.85	[1.19 – 2.88]	< 0.001
Overall survival			
SMRA (Youden LRC)	2.13	[1.58 –2.88]	< 0.001
SMA (Youden LRC)	1.53	[1.06 – 2.20]	0.02

Hazard ratios (HR) are calculated for low vs. normal/high in all models. CI, confidence interval; SMA, skeletal muscle area; SMRA, skeletal muscle radiation attenuation.

* Multivariable models were additionally adjusted for age (≤70 vs. >70 years), sex, T stage (T0–T2 vs. T3–T4), N stage (N0–N1 vs. N2–N3), and therapy (RT alone vs. CRT)

Table S3d. Cox proportional hazards models for oncological outcomes by SMA and SMRA, analyzed as dichotomous variables based on Youden-index–derived cut-offs for OS at 60 months.

	Multivariable analysis*		
Variable	HR	95% CI	p-value
Locoregional control			
SMRA (Youden OS)	1.83	[1.27–2.63]	< 0.001
SMA (Youden OS)	1.24	[0.82–1.88]	0.31
Overall survival			
SMRA (Youden OS)	2.19	[1.62–2.95]	< 0.001
SMA (Youden OS)	1.77	[1.25 –2.5]	< 0.001

Hazard ratios (HR) are calculated for low vs. normal/high in all models. CI, confidence interval; SMA, skeletal muscle area; SMRA, skeletal muscle radiation attenuation; OS, overall survival.

* Multivariable models were additionally adjusted for age (≤70 vs. >70 years), sex, T stage (T0–T2 vs. T3–T4), N stage (N0–N1 vs. N2–N3), and therapy (RT alone vs. CRT)

Table S4a. Interaction tests of SMA and SMRA (dichotomized by Youden cut-off for 60-month LRC) with sex and T stage in multivariable Cox regression for LRC.

Predictor	Interaction tested	p-value
SMA	SMA × Sex	0.28
	SMA × T stage	0.30
SMRA	SMRA × Sex	0.38
	SMRA × T stage	0.31

SMA, skeletal muscle area; SMRA, skeletal muscle radiation attenuation; LRC, locoregional control.

Table S4b. Interaction tests of SMA and SMRA (dichotomized by Youden cut-off for 60-month LRC) with sex and T stage in multivariable Cox regression for OS.

Predictor	Interaction tested	p-value
SMA	SMA × Sex	0.90
	SMA × T stage	0.02
SMRA	SMRA × Sex	0.37
	SMRA × T stage	0.50

SMA, skeletal muscle area; SMRA, skeletal muscle radiation attenuation; LRC, locoregional control; OS, overall survival.

Table S5. Cox proportional hazards models for oncologic outcomes according to SMA and SMRA (as dichotomous variables) in oropharyngeal carcinoma cases with documented HPV status (n =381).

	Multivariable analysis*		
**Variable**	**HR**	**95% CI**	**p-value**
Locoregional control			
SMRA	1.39	[0.77 – 2.53]	0.27
SMA	1.51	[0.63 – 3.66]	0.35
Overall survival			
SMRA	1.28	[0.74 –2.22]	0.37
SMA	1.72	[0.82 – 3.61]	0.15

Hazard ratios (HR) are calculated for low vs. normal/high in all models. CI, confidence interval; SMA, skeletal muscle area; SMRA, skeletal muscle radiation attenuation; HPV, human Papillomavirus.

* Multivariable models were additionally adjusted for age (≤70 vs. >70 years), sex, T stage (T0–T2 vs. T3–T4), N stage (N0–N1 vs. N2–N3), therapy (RT alone vs. CRT), and HPV status (positive vs. negative).

Data on HPV status were available for 316 of 368 oropharyngeal cases (83%).

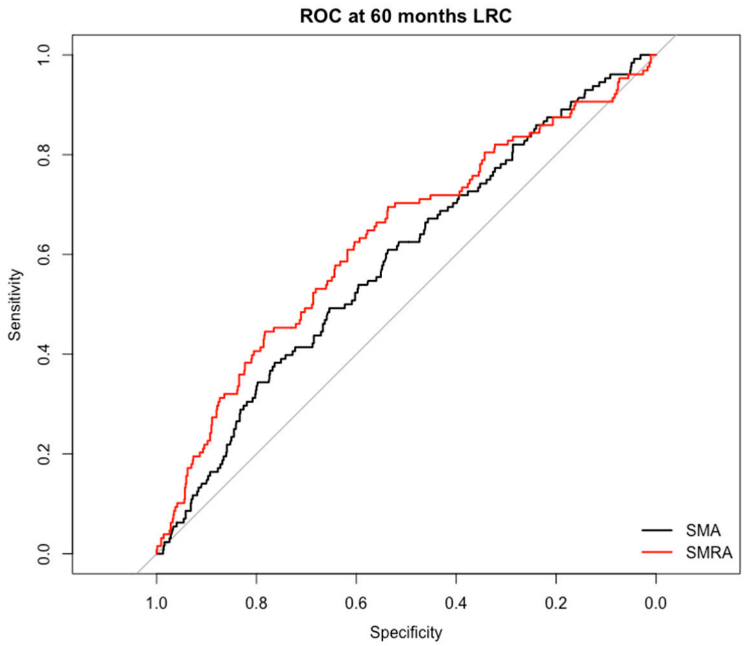

Figure S1a. Time-dependent receiver operating characteristic (ROC) curves at 60 months for skeletal muscle area (SMA, cm^2) and skeletal muscle radiation attenuation (SMRA, HU) at cervical level C3, predicting locoregional control (LRC).

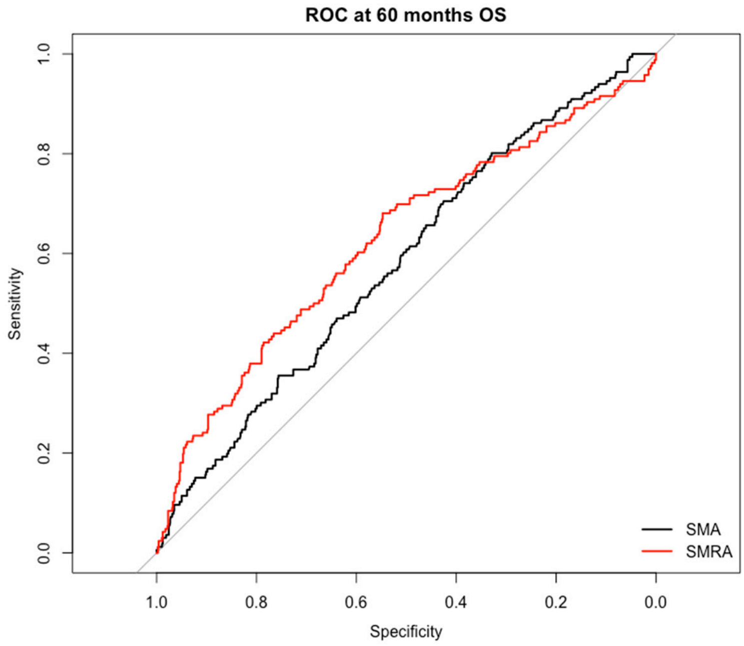

Figure S1b. Time-dependent receiver operating characteristic (ROC) curves at 60 months for skeletal muscle area (SMA, cm^2) and skeletal muscle radiation attenuation (SMRA, HU) at cervical level C3, predicting overall survival (OS).

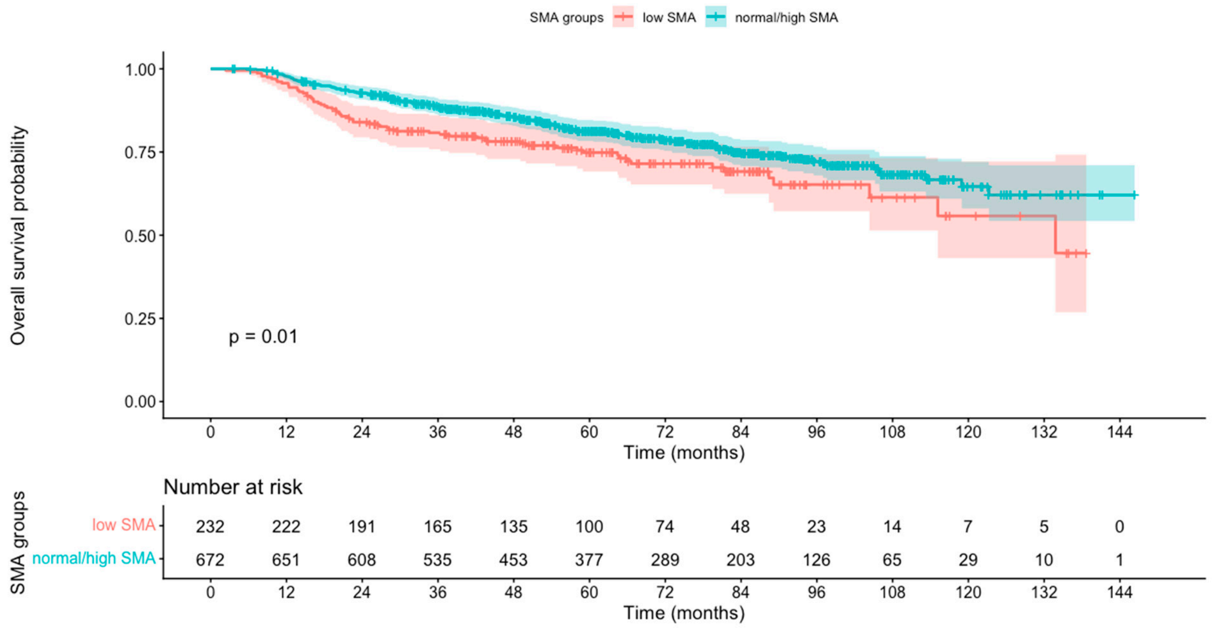

Figure S2. Kaplan-Meier survival analysis results for head and neck cancer (HNC) patients stratified by skeletal muscle area (SMA) into low and normal/high SMA groups.
